# Lichen novelties from Corvo Island (Azores, Portugal)

**DOI:** 10.3897/BDJ.12.e140418

**Published:** 2024-12-31

**Authors:** António Félix Rodrigues, Sandra I. R. Videira, André Aptroot, Rosalina Gabriel

**Affiliations:** 1 IITAA – University of the Azores, Instituto de Investigação e Tecnologias Agrárias e do Ambiente, Angra do Heroísmo, Portugal IITAA – University of the Azores, Instituto de Investigação e Tecnologias Agrárias e do Ambiente Angra do Heroísmo Portugal; 2 FGF - Fundacão Gaspar Frutuoso, Angra do Heroísmo, Portugal FGF - Fundacão Gaspar Frutuoso Angra do Heroísmo Portugal; 3 Laboratório de Botânica/Liquenologia, Instituto de Biociências, Universidade Federal de Mato Grosso do Sul, Mato Grosso do Sul, Brazil Laboratório de Botânica/Liquenologia, Instituto de Biociências, Universidade Federal de Mato Grosso do Sul Mato Grosso do Sul Brazil; 4 University of the Azores, Angra do Heroísmo, Portugal University of the Azores Angra do Heroísmo Portugal; 5 cE3c/GBA – University of the Azores, Centre for Ecology, Evolution and Environmental Changes / Azorean Biodiversity Group, Angra do Heroismo, Portugal cE3c/GBA – University of the Azores, Centre for Ecology, Evolution and Environmental Changes / Azorean Biodiversity Group Angra do Heroismo Portugal

**Keywords:** Corvo Island, remote ecosystems, Macaronesia, lichen diversity

## Abstract

**Background:**

Corvo is a small and remote island in the western group of the Azores Archipelago, Portugal. The Island's lichen biodiversity was largely understudied, with only 17 species documented to date.

**New information:**

This study reports 68 new records of lichen species on Corvo Island, representing an addition of two classes, eight orders, 18 families and 43 genera. It includes three new records for the Azores: *Acrocordiaconoidea* (Fr.) Körb., *Chrysothrixflavovirens* Tønsberg and *Glaucomariarupicola* (L.) P.F. Cannon (syn. *Lecanorarupicola* (L.) Zahlbr.). Additionally, it confirms the presence of three species previously reported in the Archipelago without specific locations: *Lecideaphaeops* Nyl., *Peltigeracanina* (L.) Willd. and *Pertusariaficorum* Zahlbr. This wealth of new lichen species records greatly enriches our understanding of biodiversity and sets a solid groundwork for upcoming ecological investigations in the Azores Archipelago.

## Introduction

Lichens are pioneers in the colonisation of rocky substrates and contribute to the formation of soil where bryophytes and, eventually, vascular plants, can grow. They are sensitive to environmental changes such as atmospheric pollution, climate change and human activity which makes them useful as key indicators of ecosystem health (Vieira et al. 2004). Despite their ecological importance, lichens have been less studied than plants, largely due to the specialised knowledge required for identifying their diverse forms and genera. The lichen identification work in the Azores has been largely reliant on visiting experts which highlights a need for local scientific engagement. The challenge of conducting in-depth studies on remote islands is often compounded by a scarcity of resources, leading to isolated efforts rather than collaborative research (Ahti and Aptroot 2009, Etayo and Berger 2009, van den Boom 2020).

The Azores Archipelago is comprised by nine islands of volcanic origin (Flores, Corvo, Pico, Faial, São Jorge, Terceira, Graciosa, São Miguel and Santa Maria) that are located in the northernmost part of the Macaronesia biogeographical region. The islands are orientated on a WNW-ESE axis spanning 615 km, approximately 1615 km west from mainland Portugal, 1935 km east from Canada and 1508 km north-west from Morocco (Google Earth Pro 2024). The North Atlantic Current, also known as Gulf Stream, creates a mild maritime climate characterised by abundant rainfall, high humidity and constant winds. Corvo Island is the northernmost, the smallest (17 km²) and the least populated island (384 people) in the Archipelago (Forjaz 2004, SREA - Serviço Regional de Estatística dos Açores 2021).

The earliest expeditions to explore the fauna and flora of Azores that included Corvo Island date back to the mid-nineteenth century. The botanists Christian Hochstetter and Karl Hochstetter visited the Archipelago in 1838. Seubert (1844) documented their efforts and listed 10 lichen species. The botanist Hewett Cottrell Watson visited the archipelago in 1842 and he reported 14 lichen species (Watson 1844). Unfortunately, neither Seubert (1844) nor Watson (1844) specified in which islands each lichen species was observed. In 1857, the malacologists Arthur Morelet and Henri Drouët visited all the islands of Azores with the exception of São Jorge. From that expedition, Drouet (1866) reported a total 40 lichen species, but only *Roccellatinctoria* D.C. was specifically mentioned as present in all islands.

Trelease (1897) is the first to report lichen species specifically from Corvo Island, namely, *Coccocarpiaerythroxyli* (Spreng.) Swinscow & Krog (as *Pannariamolybdaea*), *Ramalinacuspidata* (Ach.) Nyl., *Roccellatinctoria* D.C., *Stereocaulonazoreum* Nyl. (as *S.sphaerophoroides* Tuck.), *Usneaceratina* Ach. and *Usneahirta* (L.) Weber ex F.H. Wigg. More recently, Tehler et al. (2004) focused on *Roccella* taxonomy, based on morphological characteristics and phylogenetic placement and uses two specimens of *Roccella* from Corvo: *R.maderensis* (J.Steiner) Follmann and *R.tinctoria* D.C.. Subsequently, Clerc (2006) focused on *Usnea* taxonomy and chemistry and describes the new species *Usneasubflammea* P. Clerc with a type specimen from Pico and an additional specimen examined from Corvo. Although Tavares (1987) reported *Usneaceratina* Ach. for all Macaronesian islands, this species presence in Azores is doubtful because no specimens have been observed (Clerc 2006).

Checklists were compiled over the years on the diversity of lichens known from the Azores (Hafellner 1995, Hafellner 2008). The most recent checklist, by Aptroot et al. (2010), includes a total of 13 species for Corvo Island. In addition to the above-mentioned seven species (excluding *U.ceratina*) it includes *Parmotremareticulatum* (Taylor) M. Choisy, *Roccellaallorgei* Abbayes, *Rocellacanariensis* Darb., *Roccellaphycopsis* Ach., *Roccellatuberculata* Vain and *Nephromatangeriense* (Maheu & A.Gillet) Zahlbr. to the known species from Corvo Island.

In their most recent work, Elias et al. (2022) added five lichen novelties to Corvo Island: *Caloplacadalmatica* (A. Massal.) H.Olivier, *Leprarialobificans* Nyl., *Leucodermialeucomelos* (L.) Kalb, *Roccellographacircumscripta* (Leight.) Ertz & Tehler and *Xanthoriaaureola* (Ach.) Erichsen. At this point, the catalogue of known lichen species from Corvo Island appeared to total 18 species.

The present work aims to compile an updated list of the known lichen species from Corvo Island. We correct the initial catalogue of known species for Corvo to 17 species, based on two points: 1) both specimens from *Usneahirta* and *Usneaceratina* from Trelease (Trelease 1897) are currently identified as *Usneacornuta* (NMNH, Smithsonian Institution 2024); 2) the species *Roccellacanariensis* Darb. has been synonymised with *Roccellatinctoria* D.C. (Tehler et al. 2009).

This study reports 68 new records of lichen species on Corvo Island, representing an addition of two classes, eight orders, 18 families and 43 genera. Three species are novelties for the Azores Archipelago: *Acrocordiaconoidea* (Fr.) Körb., *Chrysothrixflavovirens* Tønsberg and *Glaucomariarupicola* (L.) P.F. Cannon (syn. *Lecanorarupicola* (L.) Zahlbr.), while three other species, previously reported in the Archipelago without specific locations, were also observed in Corvo Island: *Lecideaphaeops* Nyl., *Peltigeracanina* (L.) Willd. and *Pertusariaficorum* Zahlbr.

This effort contributes to increasing our knowledge of lichen diversity in oceanic ecosystems and will enable comparisons with other isolated islands, deepening our grasp of lichen ecology and evolution in such environments.

## General description

### Purpose

Identify and report lichen novelties in Corvo island in Azores Archipelago, based on bibliographic research, new collections and identifications.

## Project description

### Title

Lichen novelties on Corvo Island in the Azores Archipelago, Portugal

### Personnel

- Fieldwork (site selection and sample collection): António Félix Rodrigues;
- Taxonomists: António Félix Rodrigues, André Aptroot;
- Voucher specimen management: António Félix Rodrigues, Rosalina Gabriel;
- Database management: Rosalina Gabriel, Sandra Videira;
- Darwin Core databases management: Rosalina Gabriel, Sandra Videira.

### Study area description

**Figure 1.**
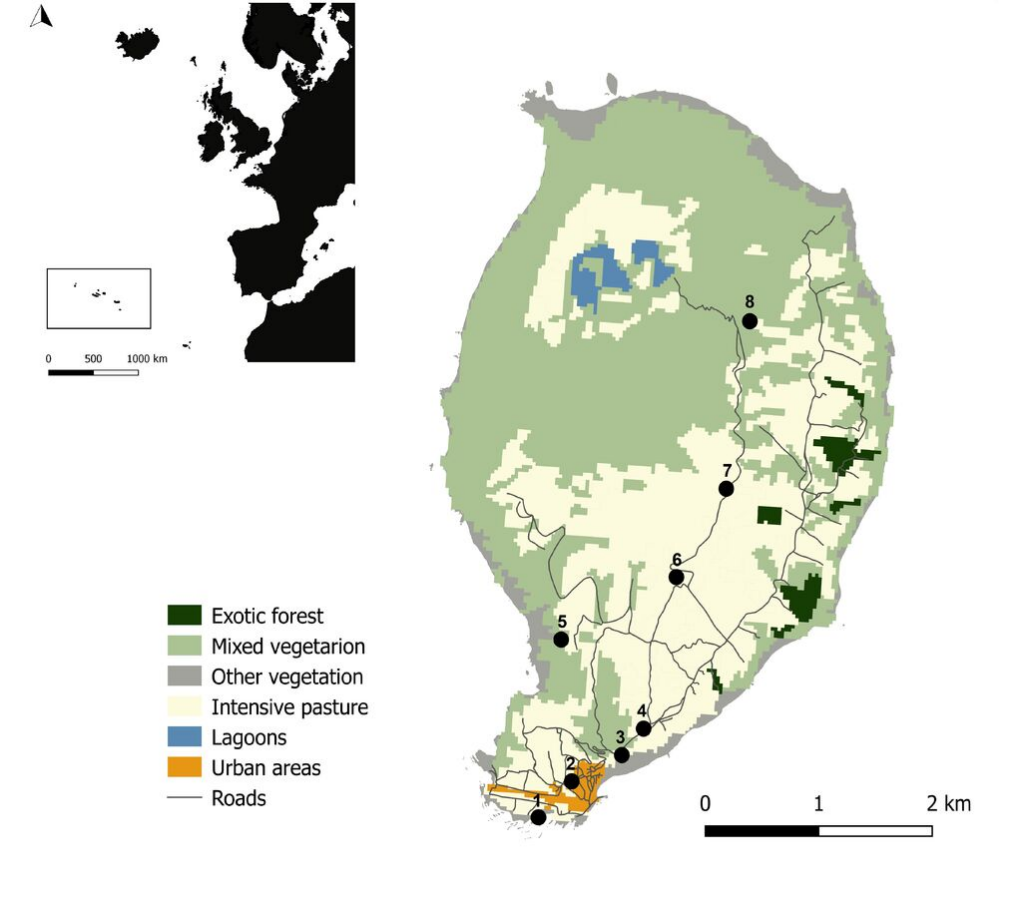
Study area location, land use and collection sites. **A** The Azores Archipelago location at approximately 1615 km west from mainland Portugal, Europe; **B** Map of Corvo Island with land-use information (data from Cruz et al. (2007)) and numbered 1 to 8, are the locations where specimens were colected that are further detailed in Table 1 (Credit: Enésima Pereira, Azorean Biodiversity Group).

Corvo is the northernmost, the smallest (17 km²) and the least populated island (384 people) in the Archipelago. The only urban centre, Vila do Corvo, is located on the southern tip of the Island, roughly at sea level. Agriculture, with walls or hedges as wind protection, is mostly limited to the coastal area surrounding the village and extending through the eastern coast. Pastureland, used for raising dairy cattle, is located north from the village and inside the Island’s caldera – “Caldeirão”. The north part of the Island is dominated by the caldera of the volcano that created the Island. It reaches 720 m in altitude and has several small lakes and islets at the bottom. The mountainous slopes are covered in mixed vegetation and no native laurel forests remain (Imber & Gygax 1971, Bussmann 2016) (Fig. 1). The caldera, plus the majority of the Island’s coastline and its islets, are part of Corvo’s Nature Park that was created in 2006 in order to conserve and protect species habitat and natural resources (Decreto Legislativo Regional 56/2006/A, Diário da República n.º 245/2006, Série I de 2006-12-22).

### Design description

Samples were collected by António Félix Rodrigues, from various locations on Corvo Island (Table 1) in June 2009 and September 2023.

### Funding

António Félix Rodrigues fieldwork in Corvo Island was funded by EDA Renováveis in 2023.

Sandra I.R. Videira is funded by Centre for Ecology, Evolution and Environmental Changes (cE3c), with base funding ref. UIDB/00329/2020-2024, DOI 10.54499/UIDB/00329/2020, Fundação para a Ciência e Tecnologia, I.P. (FCT).

Rosalina Gabriel is currently funded by FCT-UIDB/00329/2020-2024, DOI 10.54499/UIDB/00329/2020 (Thematic Line 1–integrated ecological assessment of environmental change on biodiversity) and Azores DRCT Pluriannual Funding (M1.1.A/FUNC.UI&D/010/2021-2024).

## Sampling methods

### Sampling description

Our methodology involved the collection of specimens in different locations (Table 1) and from different substrates on the Island, with detailed photographs taken to document the characteristics of each specimen. In June 2009, sampling took place during one and a half days (approximately 4 hours total). In September 2023, sampling took 3 days (approximately 12 hours total). Most samples were collected in Vila do Corvo and its surrounding areas, from walls and rocks which were easily accessible.

### Quality control

Our methodology involved thorough examination of all registered specimens using conventional techniques in the field, coupled with detailed photography to document key features. The specimens were identified by António Félix Rodrigues, while André Aptroot contributed to the accurate determination of the less common species, based on detailed photography of the specimens.

### Step description

The specimens were initially compared with photographs in works of reference (Schumm 2008, Schumm and Aptroot 2013, McMullin and Anderson 2014, Dobson 2018, Elias et al. 2022) and further studied following the methodologies described by Schumm (2008) and Schumm and Aptroot (2013) for lichens in the Azores, Madeira and Canary Islands. The species nomenclature follows Mycobank (https://www.mycobank.org), Index Fungorum (https://www.indexfungorum.org) and recently published literature that is detailed for each case in the results section (e.g. several chapters of Lichens of Great Britain and Ireland 3^rd^ edition (https://britishlichensociety.org.uk/identification/lgbi3). The species distribution of species follows the most updated information available in the AZORESBIOPORTAL (https://azoresbioportal.uac.pt) and recently published literature. The IUCN Red List status was accessed in the Consortium of Lichen Herbaria (https://lichenportal.org/portal/checklists/checklist.php?clid=1448&pid=).

## Geographic coverage

### Description

The study was conducted in Corvo Island, Azores Archipelago, Portugal.

### Coordinates

39.669 and 39.727 Latitude; -31.129 and -31.082 Longitude.

## Taxonomic coverage

### Description

Lichens

### Taxa included

**Table:** 

Rank	Scientific Name	
kingdom	Fungi	
phylum	Ascomycota	

## Temporal coverage

### Notes

Bibliographic research temporal coverage from 1844 to 2024; specimens collection temporal coverage in Corvo Island includes one day and a half in June 2009 and three days in September 2023.

## Collection data

### Collection name

AZU_Section Lichens (specimens from 2009); Personal collection of António Félix Rodrigues (specimens from 2023)

### Collection identifier

AZU; herb. António Félix Rodrigues

### Specimen preservation method

dry

### Curatorial unit

Rosalina Gabriel (AZU); Félix Rodrigues (herb. António Félix Rodrigues)

## Usage licence

### Usage licence

Creative Commons Public Domain Waiver (CC-Zero)

## Data resources

### Data package title

Lichen novelties in Corvo Island, Azores, Portugal

### Resource link

https://www.gbif.org/dataset/04f83194-eb55-4c59-8047-ffbc93c1a4e4

### Alternative identifiers

http://ipt.gbif.pt/ipt/resource?r=lichens-azo_cor_afr2024

### Number of data sets

2

### Data set 1.

#### Data set name

Event table

#### Data format

Darwin Core Archive format

#### Character set

UTF-8

#### Download URL

http://ipt.gbif.pt/ipt/resource?r=lichens-azo_cor_afr2024

#### Data format version

1.5

#### Description

The dataset was published in the Global Biodiversity Information Facility platform (GBIF). The following data table includes all the records for which a taxonomic identification of the species was possible. The dataset submitted to GBIF is structured as a sample event dataset that has been published as a Darwin Core Archive (DwCA), which is a standardised format for sharing biodiversity data as a set of one or more data tables. The core data file contains 40 records (eventID). This GBIF IPT (Integrated Publishing Toolkit, version 2.5.6) archives the data and, thus, serves as the data repository. The data and resource metadata are available for download in the Portuguese GBIF Portal IPT (Rodrigues et al. 2024).

**Data set 1.** 

Column label	Column description
eventID	Identifier of the events, unique for the dataset.
type	The nature or genre of the event.
datasetName	The name identifying the dataset from which the record was derived.
samplingProtocol	The sampling protocol used to capture the species.
eventDate	Date or date range the record was collected.
year	Year of the event.
month	Month of the event.
day	Day of the event.
Habitat	The habitat of the sample.
Continent	Name of the continent.
islandGroup	Name of archipelago.
island	Name of the island.
country	Country of the sampling site.
countryCode	ISO code of the country of the sampling site.
municipality	Municipality of the sampling sites.
locality	Name of the locality.
verbatimElevation	Description of the elevation (altitude above sea level) of the location.
decimalLatitude	Approximate centre point decimal latitude of the field site in GPS coordinates.
decimalLongitude	Approximate centre point decimal longitude of the field site in GPS coordinates.
geodeticDatum	The ellipsoid, geodetic datum or spatial reference system (SRS), upon which the geographic coordinates given in decimalLatitude and decimalLongitude are based.
coordinateUncertaintyInMetres	Uncertainty of the coordinates of the centre of the sampling plot.
georeferenceSources	Maps used to georeference the decimalLatitude and decimalLongitude.

### Data set 2.

#### Data set name

Occurrence table

#### Data format

Darwin Core Archive format

#### Character set

UTF-8

#### Download URL

http://ipt.gbif.pt/ipt/resource?r=lichens-azo_cor_afr2024

#### Data format version

1.5

#### Description

The dataset was published in the Global Biodiversity Information Facility platform (GBIF). The following data table includes all the records for which a taxonomic identification of the species was possible. The dataset submitted to GBIF is structured as a sample event dataset that has been published as a Darwin Core Archive (DwCA), which is a standardised format for sharing biodiversity data as a set of one or more data tables. The core data file contains 126 records (occurenceID). This GBIF IPT (Integrated Publishing Toolkit, version 2.5.6) archives the data and, thus, serves as the data repository. The data and resource metadata are available for download in the Portuguese GBIF Portal IPT (Rodrigues et al. 2024).

**Data set 2.** 

Column label	Column description
eventID	Identifier of the events, unique for the dataset.
licence	Reference to the licence under which the record is published.
institutionID	The identity of the institution publishing the data.
institutionCode	The code of the institution publishing the data.
datasetName	The name identifying the data-set from which the record was derived.
type	Type of the record, as defined by the Public Core standard.
basisOfRecord	The nature of the data record.
collectionCode	The code of the collection where the specimens are conserved.
catalogNumber	A unique identifier for the record within the collection.
otherCatalogNumbers	A list (concatenated and separated) of alternate fully qualified catalogue numbers for the same occurrence in other collections.
recordedBy	A list of names of people who performed the sampling in the field.
recordNumber	An identifier given to the occurrence at the time it was recorded (specimen collector's number).
occurrenceID	Identifier of the record, coded as a global unique identifier.
identifiedBy	A list of names of people who assigned the taxon to the subject.
disposition	The current state of a specimen in the collection identified in collectionCode.
taxonRank	Lowest taxonomic rank of the record.
kingdom	Kingdom name.
phylum	Phylum name.
class	Class name.
order	Order name.
family	Family name.
genus	Genus name.
specificEpithet	Specific epithet.
scientificNameAuthorship	Name of the author of the lowest taxon rank included in the record.
ScientificName	Species name.
establishmentMeans	The process of establishment of the species in the location, using a controlled vocabulary.
occurrenceRemarks	Comments or notes about the occurrence.

## Additional information

**Figure 2.**
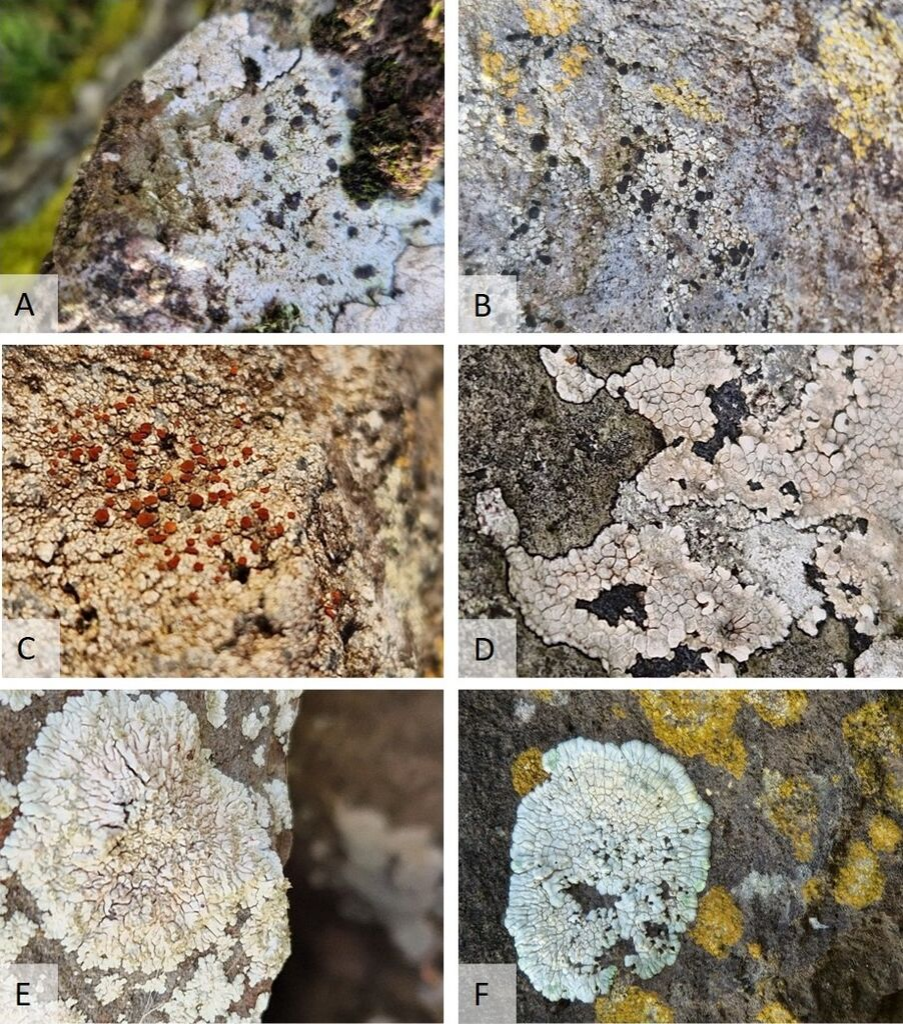
Photographs of some lichen species observed in Corvo Island. **A.** *Acrocordiagemmata*; **B.** *Buelliasubdisciformis*; **C.** *Caloplacaceracea*; **D.** *Circinariacalcarea*; **E.** *Diploiciacanescens*; **F.** *Diploiciasubcanescens*.

**Figure 3.**
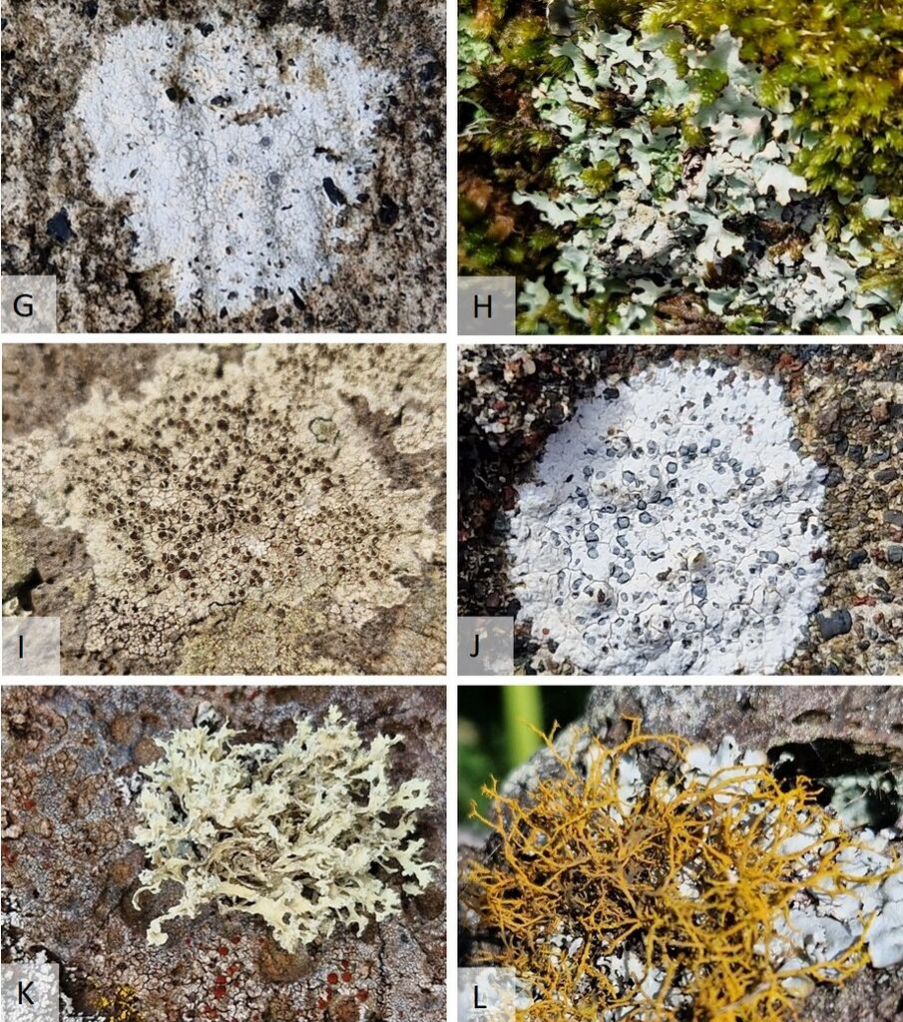
Photographs of some lichen species observed in Corvo Island. **G.** *Glaucomariarupicola*; **H.** *Hypotrachynarockii*; **I.** *Lecanoracampestris*; **J.** *Porpidiaalbocaerulescens*; **K.** *Ramalinarequienii*; **L.** *Teloschistesflavicans*.

Most lichens specimens were collected in Vila do Corvo and in the area surrounding Miradouro do Caldeirão. Most of the lichens observed had a crustose thallus and grew on basalt rock surfaces, both in urban and ruderal environments, but some were foliose or fruticose as can be seen in the example pictures below (Figs 2, 3).

The present work is the result of a significant effort in sampling the lichen biodiversity in Corvo Island and contributed greatly to increase the number species known. The lichens reported from Corvo Island belong to the phylum Ascomycota and are divided amongst 27 families. The largest family present is Teloschistaceae with seven genera and 10 species. The genus with most species observed is *Roccella*, in the Roccellaceae family, with six species (Table 2).

A total of 85 species are listed in this work, from which 79 were observed in the field. Six of the taxa listed are based only on preserved specimens in herbaria (G, NY, S and US) and literature records: *Coccocarpiaerythroxyli* (Spreng.) Swinscow & Krog, *Nephromatangeriense* (Maheu & A.Gillet) Zahlbr., *Roccellaallorgei* Abbayes, *Roccellamaderensis* (J.Steiner) Follmann, *Usneasubflammea* P.Clerc and *Usneacornuta* Körb.).

A total of three species are new to the Azores Archipelago, namely *Acrocordiaconoidea* (Fr.) Körb., *Chrysothrixflavovirens* Tønsberg and *Glaucomariarupicola* (L.) P.F. Cannon (syn. *Lecanorarupicola* (L.) Zahlbr.). Three species, previously mentioned to the Azores without a specific location, were observed in Corvo (*Lecideaphaeops* Nyl., *Peltigeracanina* (L.) Willd. and *Pertusariaficorum* Zahlbr.) (Table 3).

**Below is an annotated, alphabetically ordered list of the 85 species reported from Corvo Island.**

Species not observed in this study are marked with a ‘#’, species new to the Azores are marked with a ‘+’ and species previously known from the Azores without a specific location are marked with a ‘>’. For specimens collected in this study, the following details are provided in order: collection spot [1 to 8; cf. Table 1], location name, thallus shape, substrate, collection date and collector.

***Acarosporaumbilicata*** Bagl. 1857

Specimen examined: [**2**] Vila do Corvo, thallus crustose, on basalt rock, 25-Sep-2023, A.F. Rodrigues.

+ ***Acrocordiaconoidea*** (Fr.) Körb. 1855

Specimen examined: [**8**] Miradouro do Caldeirão, thallus crustose, on basalt rock, 26-Sep-2023, A.F. Rodrigues.

***Acrocordiagemmata*** (Ach.) A.Massal. 1854 (Fig. 2A)

Specimen examined: [**6**] Parque eólico do Cerrado das Vacas, thallus crustose, on basalt rock, 27-Sep-2023, A.F. Rodrigues.

***Amandineapunctata*** (Hoffm.) Coppins & Scheid. 1993

Specimen examined: [**2**] Vila do Corvo, thallus crustose, on *Metrosiderosexcelsa*, 27-Sep-2023, A.F. Rodrigues.

***Aquacidiaviridifarinosa*** (Coppins & P. James) Aptroot 2018 (syn. *Bacidiaviridifarinosa* Coppins & P. James 1992)

Coppins and Aptroot (2009) recognised the species *Bacidiaviridifarinosa* Coppins & P. James should be excluded from the genus *Bacidia* and assigned to the Pilocarpaceae. Aptroot et al. (2018) introduced the genus *Aquacidia* for this species and this is change is followed by Cannon et al. 2021c and Cannon et al. 2023c.

Specimen examined: [**2**] Vila do Corvo, thallus crustose, on Lapilli, 25-Sep-2023, A.F. Rodrigues.

***Bacidiaarceutina*** (Ach.) Arnold 1869

Specimen examined: [**2**] Vila do Corvo, thallus crustose, on *Metrosiderosexcelsa*, 27-Sep-2023, A.F. Rodrigues.

***Bacidialaurocerasi*** (Delise ex Duby) Zahlbr. 1926

Specimen examined: [**2**] Vila do Corvo, thallus crustose, on *Metrosiderosexcelsa*, 27-Sep-2023, A.F. Rodrigues.

***Bactrosporapatellarioides*** (Nyl.) Almq. 1869

Specimen examined: [**2**] Vila do Corvo, thallus crustose, on *Ficuscarica*, 25-Sep-2023, A.F. Rodrigues.

***Blasteniacrenularia*** (With.) Arup, Søchting & Frödén 2013 (syn. *Caloplacacrenularia* (With.) J.R. Laundon 1984)

The genus *Blastenia* is currently a separate genus from *Caloplaca* and forms a monophyletic clade in the Teloschistaceae. The combination of *Caloplacacrenularia* into *Blastenia* was proposed by Arup et al. (2013).

Specimen examined: [**2**] Vila do Corvo, thallus crustose, on basalt rock, 25-Sep-2023, A.F. Rodrigues.

***Buelliadisciformis*** (Fr.) Mudd 1861

Specimen examined: [**8**] Miradouro do Caldeirão, thallus crustose, on basalt rock, 25-Sep-2023, A.F. Rodrigues.

***Buelliasubdisciformis*** (Leight.) Jatta 1892

Specimen examined: [**2**] Vila do Corvo, thallus crustose, on basalt rock, 27-Sep-2023, A.F. Rodrigues.

***Byssolomasubdiscordans*** (Nyl.) P. James 1971 (Fig. 2B)

Specimen examined: [**6**] Parque eólico do Cerrado das Vacas, thallus crustose, on basalt rock, 26-Sep-2023, A.F. Rodrigues.

***Caloplacaceracea*** J.R. Laundon 1992 (syn. *Pyrenodesmiaceracea* (J.R. Laundon) S.Y. Kondr. 2020) (Fig. 2C)

The genus *Pyrenodesmia* was re-introduced by Arup et al. (2013) for a group of species that was separated from *Caloplaca* sensu stricto. However, the genus formed a polyphyletic clade that required further study and *Pyrenodesmia* sensu stricto included only species that lacked anthraquinones in both apothecia and thalli. Kondratyuk et al. (2020) proposed the combination *Pyrenodesmiaceracea* (J. R. Laundon) S. Y. Kondr. (Basionym: *Caloplacaceracea* J. R. Laundon 1992), but, since no molecular data are available for this species and no reason was given for the combination, we will use the previous name as in Aptroot et al. (2010).

Specimen examined: [**2**] Vila do Corvo, thallus crustose, on basalt wall, 25-Sep-2023, A.F. Rodrigues.

***Caloplacadalmatica*** (A. Massal.) H. Olivier 1909

This species was first reported from Corvo by Elias et al. (2022). The specimen of *Caloplacadalmatica* collected from Cais Velho in June 2009 was also preserved at the CBS Herbarium.

Specimens examined: [**1**] Cais Velho, thallus crustose, on basalt rock, Jun-2009, A.F. Rodrigues. [**2**] Vila do Corvo, thallus crustose, on basalt rock, 27-Sep-2023, A.F. Rodrigues. [**5**] Parque fotovoltaico do Pão de Açúcar, thallus crustose, on basalt rock, 25-Sep-2023, A.F. Rodrigues.

***Candelariellavitellina*** (Ehrh.) Müll.Arg. 1894

Specimen examined: [**2**] Vila do Corvo, thallus crustose, on basalt rock, 25-Sep-2023, A.F. Rodrigues.

+ ***Chrysothrixflavovirens*** Tønsberg 1994

This species is widely distributed in Europe and is known to occur in mainland Portugal (Rodrigues et al. 2011). In Azores, *Chrysothrixflavovirens* can be confused with *C.candelaris* which develops a brighter yellowish thallus. *Chrysothrixflavovirens* was observed colonising the bark of a *Metrosiderosexcelsa* tree in Vila do Corvo. Frisch et al. (2014) indicated that *Chrysothrix* is not monophyletic and may become segregated after thorough revision of its species.

Specimen examined: [**2**] Vila do Corvo, thallus crustose, on *Metrosiderosexcelsa*, 26-Sep-2023, A.F. Rodrigues.

***Circinariacalcarea*** (L.) A.Nordin, S.Savic & Tibell 2010 (syn. *Aspiciliacalcarea* (L.) Körb. 1859) (Fig. 2D)

The genus *Circinaria* was resurrected by Nordin et al. (2010) to include segregates from *Aspicilia* that formed a monophyletic clade and proposed the combination of *Aspiciliacalcarea* into the genus. This is currently still supported by several authors (Wijayawardene et al. 2022, Cannon et al. 2023a).

Specimen examined: [**2**] Vila do Corvo, thallus crustose, on basalt rock, 27-Sep-2023, A.F. Rodrigues.

***Cladoniafurcata*** (Huds.) Schrad. 1794

Specimen examined: [**8**] Miradouro do Caldeirão, thallus squamulose to fruticose, on basalt wall with moss, 25-Sep-2023, A.F. Rodrigues.

***Cladoniastereoclada*** Abbayes 1946

Specimen examined: [**8**] Miradouro do Caldeirão, thallus squamulose to fruticose, on soil, 25-Sep-2023, A.F. Rodrigues.

#***Coccocarpiaerythroxyli*** (Spreng.) Swinscow & Krog 1976 (syn. *Coccocarpiamolybdaea* Pers. 1827)

Initially reported from Corvo as *Coccocarpiamolybdenia* by Trelease (1897) and followed by Nylander (1898) and Navás (1909). Following the work of Arvidsson (1982), who reports the species to Faial, Aptroot et al. (2010) treated *Coccocarpiamolybdenia* as a synonym of *Coccocarpiaerythroxyli*. The species is also known from São Miguel (Henriques 1895), Corvo and Flores (Trelease 1897), Pico (Arvidsson 1990) and São Jorge (van den Boom 2020).

***Collemasubflaccidum*** Degel. 1974

Specimen examined: [**6**] Parque eólico do Cerrado das Vacas, thallus foliose, on soil, 26-Sep-2023, A.F. Rodrigues.

***Diploiciacanescens*** (Dicks.) A.Massal. 1852 (Fig. 2E)

Specimen examined: [**2**] Vila do Corvo, thallus crustose, on basalt wall, 25-Sep-2023, A.F. Rodrigues.

***Diploiciasubcanescens*** (Werner) Hafellner & Poelt 1979 (Fig. 2F)

Specimen examined: [**2**] Vila do Corvo, thallus crustose, on basalt rock, 27-Sep-2023, A.F. Rodrigues.

***Diploschistesdiacapsis*** (Ach.) Lumbsch 1988

Specimen examined: [**2**] Vila do Corvo, thallus crustose, on basalt wall, 25-Sep-2023, A.F. Rodrigues.

***Dirinamassiliensis*** Durieu & Mont. 1847

Asexual morphs described earlier at the rank forma are no longer recognised as taxonomic units (e.g. D.massiliensisf.sorediata) (Tehler et al. 2013). The sorediate morphs of *Dirina* species seem to be phylogenetically inseparable from their apothecial counterparts (Cannon et al. 2023b).

Specimen examined: [**2**] Vila do Corvo, thallus crustose, on basalt rock, Jun-2009, A.F. Rodrigues.

***Dirinariaapplanata*** (Fée) D.D.Awasthi 1970

Specimen examined: Vila do Corvo, thallus foliose, on basalt rock, Jun-2009, A.F. Rodrigues.

***Flavoparmeliacaperata*** (L.) Hale 1986

This species is recorded on all the islands, except for São Jorge (Elias et al. (2022) and it is listed as least concern in IUCN Red List (Allen et al. 2020).

Specimens examined: [**2**] Vila do Corvo, thallus foliose, on basalt wall, 27-Sep-2023, A.F. Rodrigues. [**4**] Central Termoeléctrica, thallus foliose, on basalt wall, 26-Sep-2023, A.F. Rodrigues. [**5**] Parque fotovoltaico do Pão de Açúcar, thallus foliose, on basalt wall, 25-Sep-2023, A.F. Rodrigues. [**6**] Parque eólico do Cerrado das Vacas, thallus foliose, on basalt wall, 25-Sep-2023, A.F. Rodrigues. [**7**] Baldio, ponta da Ribeira da Ponte, thallus foliose, on basalt wall, 26-Sep-2023, A.F. Rodrigues. [**8**] Miradouro do Caldeirão, thallus foliose, on basalt rock, 25-Sep-2023, A.F. Rodrigues.

***Flavoplacamarina*** (Wedd.) Arup, Frödén & Søchting 2013 (syn. *Caloplacamarina* (Wedd.) Zahlbr. 1921)

The genus *Flavoplaca* is one of the largest in the family *Teloschistaceae* and the combination of *Lecanoramarina* into the genus was proposed by Arup et al. (2013).

Specimen examined: [**2**] Vila do Corvo, thallus crustose, on basalt wall, 27-Sep-2023, A.F. Rodrigues.

+ ***Glaucomariarupicola*** (L.) P.F. Cannon 2022 (syn. *Lecanorarupicola* (L.) Zahlbr. 1928) (Fig. 3G)

*Glaucomariarupicola* is the type species of the genus *Glaucomaria*, but the combination based on the basionym *Lichenrupicola* L. was only recently formally proposed. This species phylogenetic position and morphological characterisation are well studied (Cannon et al. 2022). *Glaucomariarupicola* has a worldwide distribution (GBIF), including Portugal mainland, but was thus far unknown from the Azores. On the Island of Corvo, it grows on basaltic rocks at the summit of the Caldeirão on a sunny, exposed outcrop.

Specimen examined: [**8**] Miradouro do Caldeirão, thallus crustose, on basalt rock, 25-Sep-2023, A.F. Rodrigues.

***Gyalolechiaflavovirescens*** (Wulfen) Søchting, Frödén & Arup 2013 (syn. *Caloplacaflavovirescens* (Wulfen) Dalla Torre & Sarnth. 1902)

Until more information becomes available, we consider the genus *Gyalolechia* as defined by Arup et al. (2013) (followed by Lücking et al. (2017a), Wilk et al. (2021)) and not as proposed by Kondratyuk et al. (2014) and Kondratyuk et al. (2017). *Gyalolechiaflavovirescens* has a worldwide distribution, but was only known in Azores from Terceira (Aptroot et al. 2010). In Corvo, it is found as saxicolous on basaltic rocks.

Specimen examined: [**2**] Vila do Corvo, thallus crustose, on basalt wall, 26-Sep-2023, A.F. Rodrigues.

***Hertelianagagei* (Sm.) J.R.Laundon 2005** (syn. *Hertelianataylorii* (Salwey) P. James 1980)

*Hertelianagagei* (Sm.) J.R.Laundon is the type species of the genus *Herteliana* in Squamarinaceae (Lücking et al. 2017b). Laundon (2005) stated that the earlier name *Lichengagei* Sm. 1814 takes precedence over *Biatorataylorii* Salwei 1853 (which is also a provisional name) and proposed the name *Hertelianagagei* (Sm.) J.R.Laundon for the species. In Azores, this species is now only unknown from Graciosa Island (Aptroot et al. 2010, van den Boom 2020).

Specimen examined: [**8**] Miradouro do Caldeirão, thallus crustose, on basalt rock, 25-Sep-2023, A.F. Rodrigues.

***Hyperphysciaadglutinata*** (Flörke) H. Mayrhofer & Poelt 1979

Specimen examined: [**2**] Vila do Corvo, thallus foliose, on *Metrosiderosexcelsa*, 27-Sep-2023, A.F. Rodrigues.

***Hypotrachynacryptochlora*** (Vain.) D.Hawksw. & A.Crespo 2011 (syn. *Parmelinopsiscryptochlora* (Vain.) Elix & Hale 1987)

Specimen examined: [**2**] Vila do Corvo, thallus foliose, on basalt rock, Jun-2009, A.F. Rodrigues.

***Hypotrachynarevoluta*** (Flörke) Hale 1975

Specimen examined: [**2**] Vila do Corvo, thallus foliose, on basalt rock, 26-Sep-2023, A.F. Rodrigues.

***Hypotrachynarockii*** (Zahlbr.) Hale 1975 (Fig. 3H)

Specimen examined: [**6**] Parque eólico do Cerrado das Vacas, thallus foliose, on basalt wall with moss, 25-Sep-2023, A.F. Rodrigues.

***Ingaderiavandenboomii*** Ertz 2023 (syn. *Llimonaeasorediata* Van den Boom, M.Brand & Elix 2007)

The phylogenetic study of the genus *Llimonaea* showed it was paraphyletic and an enlarged concept of the genus *Ingaderia* was proposed. As a consequence, the species *Llimonaeasorediata* Van den Boom, M.Brand & Elix was combined into the new genus as *Ingaderiavandenboomii* Ertz (Ertz and Tehler 2023). This species was only previously known from Terceira (Aptroot et al. 2010) and Pico (van den Boom 2020) Islands.

Specimen examined: [**2**] Vila do Corvo, thallus crustose, on basalt rock, 25-Sep-2023, A.F. Rodrigues.

***Lecaniarabenhorstii*** (Hepp) Arnold 1884

Specimen examined: [**2**] Vila do Corvo, thallus crustose, on *Metrosiderosexcelsa*, 26-Sep-2023, A.F. Rodrigues.

***Lecanoracampestris*** (Schaer.) Hue 1888 (Fig.3.I.)

The species can be confused with *L.cenisia* because both species have distributions that may overlap in some areas, as seems to be the case on Corvo Island. Both species have relatively small apothecia, but *L.cenisia* generally has smaller apothecia compared to *L.campestris*, which tends to have larger apothecia, as described in literature. In the Azores, the apothecia of *L.cenisia* are often lighter (e.g. pale yellow or orange), while those of *L.campestris* can be darker or range from yellowish to light brown, depending on the environmental and growth conditions.

Specimen examined: [**2**] Vila do Corvo, thallus crustose, on basalt rock, Jun-2009, A.F. Rodrigues.

***Lecanoracenisia*** Ach. 1810

See *Lecanoracampestris*.

Specimens examined: [**2**] Vila do Corvo, thallus crustose, on basalt rock, 25-Sep-2023, A.F. Rodrigues. [**6**] Parque eólico do Cerrado das Vacas, thallus crustose, on basalt rock, 25-Sep-2023, A.F. Rodrigues.

***Lecanorachlarotera*** Nyl. 1872

Specimen examined: [**2**] Vila do Corvo, thallus crustose, on *Metrosiderosexcelsa*, 27-Sep-2023, A.F. Rodrigues.

**>*Lecideaphaeops*** Nyl. 1858

This species was previously known from Azores without a specific location (Hafellner 1995, Aptroot et al. 2010) and is now observed in Corvo Island.

Specimen examined: [**2**] Vila do Corvo, thallus crustose, on basalt rock, 25-Sep-2023, A.F. Rodrigues.

***Lecidellascabra*** (Taylor) Hertel & Leuckert 1969

Specimen examined: [**2**] Vila do Corvo, thallus crustose, on basalt rock, 25-Sep-2023, A.F. Rodrigues.

***Leprariaincana*** (L.) Ach. 1803

Specimen examined: [**2**] Vila do Corvo, thallus crustose, on basalt rock, 25-Sep-2023, A.F. Rodrigues.

***Leprarialobificans*** Nyl. 1873

This species was first reported from Corvo by Elias et al. (2022); its is present in all the islands of the Archipelago (Table 3).

Specimens examined: [**2**] Vila do Corvo, thallus crustose, on basalt rock, Jun-2009, A.F. Rodrigues. [**5**] Parque fotovoltaico do Pão de Açúcar, thallus crustose, on basalt rock, 25-Sep-2023, A.F. Rodrigues. [**6**] Parque eólico do Cerrado das Vacas, thallus crustose, on basalt rock, 26-Sep-2023, A.F. Rodrigues.

***Leprocaulonmicroscopicum*** (Vill.) Gams ex D.Hawksw. 1974

Specimens examined: [**2**] Vila do Corvo, thallus fruticose, on basalt rock, Jun-2009, A.F. Rodrigues; ibid., on basalt rock, 25-Sep-2023, A.F. Rodrigues.

***Leptogiumcyanescens*** (Ach.) Körb. 1855

Specimen examined: [**6**] Parque eólico do Cerrado das Vacas, thallus foliose, on basalt wall with moss, 25-Sep-2023, A.F. Rodrigues.

***Leucodermialeucomelos*** (L.) Kalb 2015 (syn. *Heterodermialeucomelos* (L.) Poelt 1965)

The first observation of this species in Corvo Island was reported by Elias et al. (2022).

Specimens examined: [**2**] Vila do Corvo, thallus foliose, on basalt rock, Jun-2009, A.F. Rodrigues; ibid., on basalt wall, 27-Sep-2023, A.F. Rodrigues. [**5**] Parque fotovoltaico do Pão de Açúcar, thallus foliose, on basalt wall, 25-Sep-2023, A.F. Rodrigues. [**6**] Parque eólico do Cerrado das Vacas, thallus foliose, on basalt wall, 25-Sep-2023, A.F. Rodrigues. [**7**] Baldio, ponta da Ribeira da Ponte, thallus foliose, on basalt rock, 26-Sep-2023, A.F. Rodrigues.

***Myriolecisdispersa*** (Pers.) Śliwa, Zhao Xin & Lumbsch 2015 (syn. *Lecanoradispersa* (Pers.) Sommerf. 1826)

Zhao et al. (2015) used the name *Myriolecis* to accommodate the monophyletic *Lecanoradispersa* species group and proposed the combination of *Lecanoradispersa* into the genus. Kondratyuk et al. (2019) claimed that *Polyozosia* A. Massal. 1855 has priority over *Myriolecis* Clem. 1909, but the genus and species name changes proposed were considered superfluous (Cannon et al. 2022) and, thus, we will use the name *Myriolecisdispersa* for this species. It has been observed in six islands in Azores (Table 3).

Specimen examined: [**2**] Vila do Corvo, thallus crustose, on *Metrosiderosexcelsa*, 26-Sep-2023, A.F. Rodrigues.

***Myriolecispoliophaea*** (Wahlenb.) P.F. Cannon 2022 (syn. *Lecanorapoliophaea* (Wahlenb.) Ach. 1810)

This species has only recently been sequenced (Kistenich et al. 2018) and combined into *Myriolecis* (Cannon et al. 2022). It has been observed in only two islands, Corvo and Terceira (Table 3).

Specimen examined: [**2**] Vila do Corvo, thallus crustose, on basalt rock, 26-Sep-2023, A.F. Rodrigues.

#***Nephromatangeriense*** (Maheu & A.Gillet) Zahlbr. 1932

*Nephromatangeriense* (Maheu & A.Gillet) Zahlbr. is indicated in Aptroot et al. (2010) for Corvo. There is a specimen collected by Magnus Fries in 1965 in Corvo and identified by Arvidsson in 1991 (in Herbarium S). Magnus Fries did indeed visit Azores in 1965 (Fries 1968) to collect sediments from crater lakes. Apparently, he also collected a handful of specimens of lichens from a few of the islands (see Consortium Lichen Herbarium).

***Ochrolechiaparella*** (L.) A.Massal. 1852

Specimen examined: [**2**] Vila do Corvo, thallus crustose, on basalt rock, Jun-2009, A.F. Rodrigues.

***Paralecanographagrumulosa*** (Dufour) Ertz & Tehler 2011 (syn. *Lecanographagrumulosa* (Dufour) Egea & Torrente 1994)

*Paralecanographa* is a single species genus created by Ertz and Tehler (2011). It is occasionaly a lichenicolous lichen, initially parasitic on thalli of *Dirina* and *Roccella*, but later developing an independent thallus (Diederich et al. 2018, Cannon et al. 2021a). In Corvo Island, *P.grumulosa* was observed parasitising a *Rocellaphycopsis* specimen growing on a house with basaltic walls in the village. The species was previously known only from São Miguel (Aptroot et al. 2010) and Terceira (specimens collected by Anders Tehlers in 2010 (deposited in herbarium S)).

Specimen examined: [**2**] Vila do Corvo, thallus crustose, on *Rocellaphycopsis*, 27-Sep-2023, A.F. Rodrigues.

***Parmotremacrinitum*** (Ach.) M. Choisy 1952

Specimens examined: [**6**] Parque eólico do Cerrado das Vacas, thallus foliose, on basalt wall, 26-Sep-2023, A.F. Rodrigues. [**7**] Baldio, ponta da Ribeira da Ponte, thallus foliose, on basalt wall, 26-Sep-2023, A.F. Rodrigues.

***Parmotremaperlatum*** (Huds.) M.Choisy 1952

Specimen examined: [**8**] Miradouro do Caldeirão, thallus foliose, on basalt rock, 25-Sep-2023, A.F. Rodrigues.

***Parmotremareticulatum*** (Taylor) M.Choisy 1952

Specimens examined: [**2**] Vila do Corvo, thallus foliose, on basalt rock, Jun-2009, A.F. Rodrigues. [**5**] Parque fotovoltaico do Pão de Açúcar, thallus foliose, on basalt rock, 25-Sep-2023, A.F. Rodrigues. [**6**] Parque eólico do Cerrado das Vacas, thallus foliose, on basalt rock, 26-Sep-2023, A.F. Rodrigues. [**7**] Baldio, ponta da Ribeira da Ponte, thallus foliose, on basalt rock, 26-Sep-2023, A.F. Rodrigues. [**8**] Miradouro do Caldeirão, thallus foliose, on basalt rock, 25-Sep-2023, A.F. Rodrigues.

**>*Peltigeracanina*** (L.) Willd. 1787

Hafellner (1995) and, subsequently, Aptroot et al. (2010) mention the existence of the species in the Azores without specifying the island or location of its occurrence. Therefore, this constitutes the first recorded location of the species in the Azores. On the Island of Corvo, it grows on the ground, amongst grass, in a shaded area, at the summit of the Caldeirão.

Specimen examined: [**8**] Miradouro do Caldeirão, thallus foliose, on soil, 25-Sep-2023, A.F. Rodrigues.

**>*Pertusariaficorum*** Zahlbr. 1914

Hafellner (2002) and, subsequently, Aptroot et al. (2010) simply mention the existence of the species in the Azores Archipelago. It is now specifically known from Corvo Island.

Specimen examined: [**2**] Vila do Corvo, thallus crustose, on *Metrosiderosexcelsa*, 26-Sep-2023, A.F. Rodrigues.

***Physciaadscendens*** H.Olivier 1882

Specimen examined: [**2**] Vila do Corvo, thallus foliose, on *Metrosiderosexcelsa*, 27-Sep-2023, A.F. Rodrigues.

***Physciacaesia*** (Hoffm.) Hampe ex Fürnr. 1839

Specimen examined: [**2**] Vila do Corvo, thallus foliose, on basalt rock, 27-Sep-2023, A.F. Rodrigues.

***Porpidiaalbocaerulescens*** (Wulfen) Hertel & Knoph. 1984 (Fig. 3J)

This species seems to be rare as it is previously only known from São Miguel (Trelease 1897, Aptroot et al. 2010). It grows on the basalt rock walls in the village.

Specimen examined: [**2**] Vila do Corvo, thallus crustose, on basalt wall, 25-Sep-2023, A.F. Rodrigues.

***Porpidiacontraponenda*** (Arnold) Knoph & Hertel 1984

Specimen examined: [**8**] Miradouro do Caldeirão, thallus crustose, on basalt rock, 25-Sep-2023, A.F. Rodrigues.

***Ramalinacanariensis*** J.Steiner 1904

Specimen examined: [**2**] Vila do Corvo, thallus fruticose, on *Metrosiderosexcelsa*, 27-Sep-2023, A.F. Rodrigues.

***Ramalinacuspidata*** (Ach.) Nyl. 1870

This species was first cited for Corvo by Trelease (1897) and followed by Nylander (1898), Navás (1909) and Aptroot et al. (2010).

Specimen examined: [**2**] Vila do Corvo, thallus fruticose, on basalt wall, 27-Sep-2023, A.F. Rodrigues.

***Ramalinadecipiens*** Mont. 1840

Specimen examined: [**2**] Vila do Corvo, thallus fruticose, on *Metrosiderosexcelsa*, 27-Sep-2023, A.F. Rodrigues.

***Ramalinarequienii*** (De Not.) Jatta 1892 (Fig. 3K)

Specimens examined: [**2**] Vila do Corvo, thallus fruticose, on basalt rock, Jun-2009, A.F. Rodrigues. [**3**] Miradouro do Portão, thallus fruticose, on basalt rock, 25-Sep-2023, A.F. Rodrigues. [**5**] Parque fotovoltaico do Pão de Açúcar, thallus fruticose, on basalt rock, 25-Sep-2023, A.F. Rodrigues.

***Ramalinasiliquosa*** (Huds.) A.L.Sm. 1918

Specimen examined: [**2**] Vila do Corvo, thallus fruticose, on basalt rock, Jun-2009, A.F. Rodrigues.

#***Roccellaallorgei*** Abbayes 1947

This species was first recorded in Corvo Island by Tehler et al. (2004) and later included in the Azores checklist by Aptroot et al. (2010).

***Roccellafuciformis*** (L.) DC. 1805

Although *Roccellafuciformis* was known from several islands in Azores, it was not recorded from Corvo Island in Aptroot et al. (2010). However, a specimen of *R.fuciformis* collected on Corvo Island was located in the Herbarium US catalogue (US-04420189, 29 July 1984, V.A.Funk & W.G.Nelson, 6337) that precedes the present observation.

Specimen examined: [**2**] Vila do Corvo, thallus fruticose, on basalt rock, Jun-2009, A.F. Rodrigues.

#***Roccellamaderensis*** (J.Steiner) Follmann 1993

Tehler et al. (2004) was the first to observe this species in Corvo Island and Aptroot et al. (2010) included it in the Azores checklist.

***Roccellaphycopsis*** (Ach.) Ach. 1810

This species was first recorded in Corvo Island by Tehler et al. (2004) and later included in the Azores checklist by Aptroot et al. (2010).

Specimens examined: [**2**] Vila do Corvo, thallus fruticose, on basalt rock, Jun-2009, A.F. Rodrigues; ibid., on basalt rock, 26-Sep-2023, A.F. Rodrigues.

***Roccellatinctoria*** DC. 1805

Trelease was the first to record this species in Corvo Island. Tehler et al. (2004) and Elias et al. (2022) later also observed this species in Corvo Island and it is currently known from all islands of the Archipelago (Aptroot et al. 2010). The species *Roccellacanariensis* Darb., listed as present in Corvo by Aptroot et al. (2010), is currently synonymised with *Roccellatinctoria* DC. (Tehler et al. 2009). Drouet (1866) highlighted the economic importance of *R.tinctoria* by describing how he observed men perilously hanging from ropes over rock cliffs by the sea (in Santa Maria) collecting this lichen mainly to export to England where it was used to dye army equipment. Despite the past economic significance of *Roccella* lichens on Corvo, most of its residents lack familiarity with the species, pointing to a gap in local knowledge and the potential for educational outreach.

Specimens examined: [**2**] Vila do Corvo, thallus fruticose, on basalt rock, Jun-2009, A.F. Rodrigues. [**3**] Miradouro do Portão, thallus fruticose, on basalt rock, 25-Sep-2023, A.F. Rodrigues.

***Roccellatuberculata*** Vain. 1901

This species was first recorded in Corvo Island by Tehler et al. (2004) and later included in the Azores checklist by Aptroot et al. (2010).

Specimen examined: [**3**] Miradouro do Portão, thallus fruticose, on basalt rock, 25-Sep-2023, A.F. Rodrigues.

***Roccellographacircumscripta*** (Leight.) Ertz & Tehler. 2011 (syn. *Peterjamesiacircumscripta* (Leight.) Ertz & Tehler 2011)

This species was first recorded in Corvo Island by Elias et al. (2022) and it is present in all islands of the Archipelago (Table 3).

Specimens examined: [**2**] Vila do Corvo, thallus crustose, on basalt rock, Jun-2009, A.F. Rodrigues; ibid., on basalt rock, 25-Sep-2023, A.F. Rodrigues. [**5**] Parque fotovoltaico do Pão de Açúcar, thallus crustose, on basalt rock, 25-Sep-2023, A.F. Rodrigues. [**8**] Miradouro do Caldeirão, thallus crustose, on basalt rock, 25-Sep-2023, A.F. Rodrigues.

***Stereocaulonazoreum*** Nyl. 1857 (previously cited as *Stereocaulonsphaerophoroides*)

The first reports of this species for Corvo Island were made by Trelease (1897), Nylander (1898) and Navás (1909) as *Stereocaulonsphaerophoroides* Tuck.. According to Lamb (1977), these earlier reports were incorrectly named *Stereocaulonsphaerophoroides* Tuck. and the correct name is *Stereocaulonazoreum* (Schaer.) Nyl. The species *Stereocaulonazoreum* (Schaer.) Nyl. is present in Azores, Madeira and the Canary Islands. Presently, the species *Stereocaulonsphaerophoroides* is a synonym of *Stereocaulonvirgatum* Ach., a species that occurs in the West Indies and Central America. The species *S.azoreum* is listed in the Azores lichen checklist by Aptroot et al. (2010) and has been recently observed in Corvo Island by Elias et al. (2022).

Specimen examined: [**8**] Miradouro do Caldeirão, thallus fruticose, on basalt rock, 25-Sep-2023, A.F. Rodrigues.

***Stereocaulonvesuvianum*** Pers. 1810

Specimen examined: [**6**] Parque eólico do Cerrado das Vacas, thallus fruticose, on basalt rock, 26-Sep-2023, A.F. Rodrigues.

***Teloschistesflavicans*** (Sw.) Norman 1852 (Fig. 3L)

This common species in Azores is now known from all the islands (Table 3). In Corvo, it was observed growing on the walls of basaltic stone over *Parmotremareticulatum* and *Leucodermialeucomelos*.

Specimens examined: [**2**] Vila do Corvo, thallus fruticose, on basalt rock, Jun-2009, A.F. Rodrigues. [**6**] Parque eólico do Cerrado das Vacas, thallus fruticose, on basalt wall over *Parmotremareticulatum* and *Heterodermialeucomelos*, 25-Sep-2023, A.F. Rodrigues.

***Trapeliainvoluta*** (Taylor) Hertel. 1973

Earlier records from Azores were determined as *Trapeliainvoluta*, but Aptroot et al. (2010) checklist placed them under *T.glebulosa*, following Laundon (2005) synonymisation. Recently, van den Boom (2016) recorded *T.glebulosa* for Terceira and mentioned it was only previously known from Faial (Gabriel 2008) which refers to the specimen from Purvis and James (1993) recorded as *T.involuta*. The species *Trapeliainvoluta* and *Trapeliaglebulosa* are not synonyms, but are closely related and easily confused. Morphologically, they overlap in size, but they can be separated based on TLC (Orange 2018, Orange et al. 2021). Phylogenetically, the genus is polyphyletic and a broad species concept has been in use until recently. The recent separation of *Gallowayiopsis* (Kondratyuk et al. 2022) and combination of *T.glebulosa* as *Gallowayiopsisglebulosa* (Sm.) S. Y. Kondr. may be premature and, until more information is available, we will follow the broader concept of *Trapelia* as in Orange et al. (2021). The Azorean specimens should be re-examined and their distribution in the Archipelago reassessed.

Specimen examined: [**8**] Miradouro do Caldeirão, thallus crustose, on basalt rock, 25-Sep-2023, A.F. Rodrigues.

***Trapeliaplacodioides*** Coppins & P. James 1984

Specimen examined: [**8**] Miradouro do Caldeirão, thallus crustose, on basalt rock, 24-Sep-2023, A.F. Rodrigues.

#***Usneaceratina*** Ach. 1910

The earlier records of *Usneaceratina* from Corvo are from Trelease (1897), Nylander (1898) and Navás (1909). The specimen colected by Trelease is currently identified as *Usneacornuta* Körb. (Herbarium US) and the specimen observed by both Nylander and Navás that was collected by B.T. Carreiro could not be located. Tavares (1952) indicated *U.ceratina* is present in all Macaronesian islands, except Cabo Verde islands. However, both Clerc (2006) and Aptroot et al. (2010) considered this a doubtful species for Azores.

#***Usneacornuta*** Körb. 1865

This species was not observed in the field in the present work and is not listed as present in Corvo by Aptroot et al. (2010). The two specimens colected in Corvo by Trelease, initially identified as *Usneaceratina* and *Usneahirta* that are stored in the US herbarium, are currently identified as *Usneainflata* (Duby) Motyka, which is a synonym of *Usneacornuta* Körb. The species is added to the list of species known from Corvo until further information is available.

#***Usneahirta*** (L.) Weber ex F.H. Wigg. 1780

*Usneahirta* was first cited by Trelease (1897) for Corvo, while Tavares (1952) reported it from the Azores Archipelago without a specific location. Clerc (2006) indicated it is a rare species in Macaronesia and considered its presence in Azores doubtful since he did not see any specimens, but Aptroot et al. (2010) indicated its presence in Corvo. Since no specimen of *Usneahirta* from Corvo could be traced and it was not observed in the field, we consider the presence of this species doubtful in Corvo Island until further evidence is gathered.

#***Usneasubflammea*** P. Clerc 2006

Clerc (2006) described this new species *Usneasubflammea* P. Clerc with a type specimen from Pico (on *Juniperusbrevifolia*) and additional specimens examined from Corvo (on *Ericaazorica*), Faial (on *Ericaazorica*) and Terceira (in mixed forests with mature endemic plant species such as: *Juniperusbrevifolia*, *Ericaazorica*, *Vacciniumcylindraceum*, *Laurusazorica* and *Ilexazorica*). This species is included in the Azores Lichen checklist by Aptroot et al. (2010).

***Varicellarialactea*** (L.) I.Schmitt & Lumbsch 2012 (syn. *Pertusarialactea* (L.) Arnold 1872)

The genus *Varicellaria* was circumscribed by Schmitt et al. (2012) who also proposed the combination of *Varicellarialactea* that is still currently in use (Cannon et al. 2021b).

Specimen examined: [**8**] Miradouro do Caldeirão, thallus crustose, on basalt rock, 25-Sep-2023, A.F. Rodrigues.

***Variosporaaurantia*** (Pers.) Arup, Frödén & Søchting 2013 (syn. *Caloplacaaurantia* (Pers.) Hellb. 1890)

Arup et al. (2013) recognised the genus *Variospora*, including both lobate and crustose members and a large variation in spore morphology. Phylogenetically, *Variospora* is divided into two subclades, one corresponding to the former *C.velana* group and the other to the former *C.aurantia* group. Kondratyuk et al. (2017) separated the subclade from the former *C.aurantia* group (including *V.aurantia* and *V.flavescens*) into the new genus *Klauderuiella*. However, the genus *Klauderuiella* has not been recognised in Wijayawardene et al. (2022) and we will use *Variospora* as in Arup et al. (2013) until further information is available.

Specimen examined: [**2**] Vila do Corvo, thallus crustose, on basalt rock, 25-Sep-2023, A.F. Rodrigues.

***Variosporaflavescens*** (Huds.) Arup, Frödén & Søchting 2013 (syn. *Caloplacaflavescens* (Huds.) J.R. Laundon 1984)

See *Variosporaaurantia*.

Specimen examined: [**2**] Vila do Corvo, thallus crustose, on basalt rock, 25-Sep-2023, A.F. Rodrigues.

***Xanthoriaaureola*** (Ach.) Erichsen 1930 (see also *Xanthoriaectaneoides* (Nyl.) Zahlbr. 1931)

Recently cited by Elias et al. (2022) as present in all Azorean islands, this species is very abundant on Corvo, particularly in coastal areas on basaltic rock. Lindblom and Ekman (2005) synonymised *Xanthoriaectaneoides* (Nyl.) Zahlbr. with *X.aureola* (Ach.) Erichsen and Aptroot et al. (2010) followed that synonymy. Arup et al. (2013) indicated that the taxonomy of *X.aureola* needed further investigations. Khodosovtsev et al. (2023) phylogenetic studies used specimens of *X.ectaneoides* from the Canary Islands that clustered apart from *X.aureola*, which suggests a different species may occur in Macaronesia. Kondratyuk et al. (2024) indicated that *X.aureola* and *X.ectaneoides* can be separated, based on phylogeny and morphology, but no specimens from Azores were observed. Until the existing specimens from Azores are re-examined or new ones collected for further studies, it makes sense to continue treating them as *X.aureola*.

Specimen examined: [**2**] Vila do Corvo, thallus foliose, on basalt rock, Jun-2009, A.F. Rodrigues.

***Xanthoriaparietina*** (L.) Th.Fr. 1860

This species is very common in Azores and is now only not recorded from Flores (Table 3).

Specimen examined: [**2**] Vila do Corvo, thallus foliose, on *Metrosiderosexcelsa*, 27-Sep-2023, A.F. Rodrigues.

## Figures and Tables

**Table 1.** List of locations where specimens were collected by António Félix Rodrigues in Corvo Island.

Collection Spot	Location name	Altitude (m)	Latitude (N)	Longitude (W)
1	Cais Velho	5	39.670000	-31.116667
2	Vila do Corvo	40	39.672871	-31.113426
3	Miradouro do Portão	100	39.675065	-31.108402
4	Central Termoeléctrica	190	39.677166	-31.106288
5	Parque fotovoltaico do Pão de Açúcar	200	39.683687	-31.115143
6	Parque eólico do Cerrado das Vacas	260	39.688889	-31.103611
7	Baldio, ponta da Ribeira da Ponte	440	39.695833	-31.098889
8	Miradouro do Caldeirão	560	39.708753	-31.097231

**Table 2.** Taxonomic categories of species reported from Corvo Island (Azores, Portugal). The species known to Corvo Island from literature before the present study are marked with an asterisk in superscript (*).

**phylum**	**class**	**order**	**family**	**genus**	**species**
Ascomycota	Arthoniomycetes*	Arthoniales*	Chrysotrichaceae	*Chrysothrix*	*flavovirens*
			incertae sedis	*Bactrospora*	*patellarioides*
			Opegraphaceae	*Ingaderia*	*vandenboomii*
				*Paralecanographa*	*grumulosa*
			Roccellaceae*	*Dirina*	*massiliensis*
				Roccella*	allorgei*
					*fuciformis*
					maderensis*
					phycopsis*
					tinctoria*
					tuberculata*
			Roccellographaceae*	Roccellographa*	circumscripta*
	Candelariomycetes	Candelariales	Candelariaceae	*Candelariella*	*vitellina*
	Dothideomycetes	Monoblastiales	Monoblastiaceae	*Acrocordia*	*conoidea*
					*gemmata*
	Lecanoromycetes*	Acarosporales	Acarosporaceae	*Acarospora*	*umbilicata*
		Baeomycetales	Trapeliaceae	*Trapelia*	*involuta*
					*placodioides*
		Caliciales*	Caliciaceae	*Amandinea*	*punctata*
				*Buellia*	*disciformis*
					*subdisciformis*
				*Diploicia*	*canescens*
					*subcanescens*
				*Dirinaria*	*applanata*
			Physciaceae*	*Hyperphyscia*	*adglutinata*
				Leucodermia*	leucomelos*
				*Physcia*	*adscendens*
					*caesia*
		Lecanorales*	Cladoniaceae*	*Cladonia*	*furcata*
					*stereoclada*
				*Herteliana*	*gagei*
				Lepraria*	*incana*
					lobificans*
				Stereocaulon*	azoreum*
					*vesuvianum*
			Lecanoraceae	*Glaucomaria*	*rupicola*
				*Lecanora*	*campestris*
					*cenisia*
					*chlarotera*
				*Lecidella*	*scabra*
				*Myriolecis*	*dispersa*
					*poliophaea*
			Parmeliaceae*	*Flavoparmelia*	*caperata*
				*Hypotrachyna*	*cryptochlora*
					*revoluta*
					*rockii*
				Parmotrema*	*crinitum*
					*perlatum*
					reticulatum*
				Usnea*	cornuta*
					subflammea*
			Roccellaceae	*Aquacidia*	*viridifarinosa*
				*Byssoloma*	*subdiscordans*
			Ramalinaceae*	*Bacidia*	*arceutina*
					*laurocerasi*
				*Lecania*	*rabenhorstii*
				Ramalina*	*canariensis*
					cuspidata*
					*decipiens*
					*requienii*
					*siliquosa*
		Lecideales	Lecideaceae	*Lecidea*	*phaeops*
				*Porpidia*	*albocaerulescens*
					*contraponenda*
		Leprocaulales	Leprocaulaceae	*Leprocaulon*	*microscopicum*
		Ostropales	Graphidaceae	*Diploschistes*	*diacapsis*
		Peltigerales*	Coccocarpiaceae*	Coccocarpia*	erythroxyli*
			Collemataceae	*Collema*	*subflaccidum*
				*Leptogium*	*cyanescens*
			Peltigeraceae*	Nephroma*	tangeriense*
				*Peltigera*	*canina*
		Pertusariales	Megasporaceae	*Circinaria*	*calcarea*
			Ochrolechiaceae	*Ochrolechia*	*parella*
			Pertusariaceae	*Pertusaria*	*ficorum*
			Varicellariaceae	*Varicellaria*	*lactea*
		Teloschistales*	Teloschistaceae*	*Blastenia*	*crenularia*
				Caloplaca*	*ceracea*
					dalmatica*
				*Flavoplaca*	*marina*
				*Gyalolechia*	*flavovirescens*
				*Teloschistes*	*flavicans*
				*Variospora*	*aurantia*
					*flavescens*
				Xanthoria*	aureola*
					*parietina*
**Total**	**4**	**13**	**27**	**55**	**85**
**NEW**	**2**	**8**	**18**	**43**	**68**

**Table 3.** Lichen species known to Corvo Island and their distribution in the Archipelago, based on bibliographic research and the present work, including species name and presence on the nine Azores islands (COR – Corvo; FLO – Flores; FAI – Faial; PIC – Pico; GRA – Graciosa; SJO – São Jorge; TER – Terceira; SMG – São Miguel; SMR – Santa Maria). Species known to Corvo before the present work are marked with an asterisk (COR*).

Species name	COR	FLO	FAI	PIC	GRA	SJO	TER	SMG	SMR
***Acarosporaumbilicata*** Bagl.	COR		FAI			SJO	TER		SMR
***Acrocordiaconoidea*** (Fr.) Körb.	COR								
***Acrocordiagemmata*** (Ach.) A.Massal.	COR						TER	SMG	
***Amandineapunctata*** (Hoffm.) Coppins & Scheid.	COR		FAI	PIC	GRA	SJO	TER	SMG	SMR
***Aquacidiaviridifarinosa*** (Coppins & P. James) Aptroot 2018	COR						TER		
***Bacidiaarceutina*** (Ach.) Arnold	COR			PIC	GRA	SJO	TER	SMG	SMR
*Bacidialaurocerasi* (Delise ex Duby) Zahlbr.	COR		FAI	PIC	GRA	SJO	TER	SMG	SMR
*Bactrosporapatellarioides* (Nyl.) Almq.	COR		FAI						
*Blasteniacrenularia* (With.) Arup, Søchting & Frödén	COR		FAI		GRA	SJO	TER	SMG	SMR
*Buelliadisciformis* (Fr.) Mudd	COR						TER		
*Buelliasubdisciformis* (Leight.) Jatta	COR					SJO		SMG	
***Byssoloma******subdiscordans*** (Nyl.) P.James	COR	FLO	FAI	PIC		SJO	TER	SMG	SMR
*Caloplacaceracea* J.R. Laundon	COR						TER		
*Caloplacadalmatica* (A. Massal.) H.Olivier	COR*	FLO	FAI	PIC	GRA	SJO	TER	SMG	SMR
***Candelariellavitellina*** (Ehrh.) Müll.Arg.	COR		FAI		GRA		TER	SMG	SMR
***Chrysothrixflavovirens*** Tønsberg 1994	COR								
***Circinariacalcarea*** (L.) A.Nordin, S.Savic & Tibell	COR						TER		
*Cladoniafurcata* (Huds.) Schrad.	COR		FAI			SJO	TER	SMG	
*Cladoniastereoclada* Abbayes	COR	FLO	FAI	PIC	GRA	SJO	TER	SMG	SMR
*Coccocarpiaerythroxyli* (Spreng.) Swinscow & Krog	COR*	FLO	FAI	PIC		SJO		SMG	
*Collemasubflaccidum* Degel.	COR		FAI	PIC	GRA		TER	SMG	
*Diploiciacanescens* (Dicks.) A.Massal.	COR		FAI	PIC	GRA		TER	SMG	SMR
*Diploiciasubcanescens* (Werner) Hafellner & Poelt	COR		FAI				TER		
*Diploschistesdiacapsis* (Ach.) Lumbsch	COR		FAI					SMG	
***Dirinamassiliensis Durieu*** & Mont.	COR				GRA	SJO	TER	SMG	
*Dirinariaapplanata* (Fée) D.D.Awasthi	COR		FAI	PIC	GRA		TER	SMG	SMR
*Flavoparmeliacaperata* (L.) Hale	COR	FLO	FAI	PIC	GRA		TER	SMG	SMR
*Flavoplacamarina* (Wedd.) Arup, Frödén & Søchting	COR			PIC		SJO	TER	SMG	SMR
*Glaucomariarupicola* (L.) P.F. Cannon	COR								
*Gyalolechiaflavovirescens* (Wulfen) Søchting, Frödén & Arup 2013	COR						TER		
*Hertelianagagei* (Sm.) J.R.Laundon 2005	COR	FLO	FAI	PIC		SJO	TER	SMG	SMR
*Hyperphysciaadglutinata* (Flörke) H.Mayrhofer & Poelt	COR		FAI	PIC	GRA	SJO	TER	SMG	SMR
*Hypotrachynacryptochlora* (Vain.) D.Hawksw. & A.Crespo	COR			PIC				SMG	
*Hypotrachynarevoluta* (Flörke) Hale	COR							SMG	SMR
*Hypotrachynarockii* (Zahlbr.) Hale 1975	COR		FAI	PIC		SJO	TER	SMG	SMR
*Ingaderiavandenboomii* Ertz 2023	COR			PIC			TER		SMR
*Lecaniarabenhorstii* (Hepp) Arnold 1884	COR						TER		
*Lecanoracampestris* (Schaer.) Hue	COR						TER	SMG	
*Lecanoracenisia* Ach.	COR		FAI		GRA		TER	SMG	
*Lecanorachlarotera* Nyl.	COR	FLO	FAI	PIC	GRA	SJO	TER	SMG	SMR
*Lecideaphaeops* Nyl.	COR								
*Lecidellascabra* (Taylor) Hertel & Leuckert	COR			PIC	GRA		TER	SMG	
*Leprariaincana* (L.) Ach.	COR	FLO		PIC			TER	SMG	SMR
*Leprarialobificans* Nyl.	COR*	FLO	FAI	PIC	GRA	SJO	TER	SMG	SMR
*Leprocaulonmicroscopicum* (Vill.) Gams ex D.Hawksw.	COR		FAI	PIC	GRA		TER	SMG	SMR
*Leptogiumcyanescens* (Ach.) Körb.	COR	FLO	FAI	PIC	GRA		TER	SMG	SMR
*Leucodermialeucomelos* (L.) Kalb	COR*	FLO	FAI	PIC	GRA	SJO	TER	SMG	SMR
*Myriolecisdispersa* (Pers.) Śliwa, Zhao Xin & Lumbsch	COR		FAI		GRA		TER	SMG	SMR
*Myriolecispoliophaea* (Wahlenb.) P.F.Cannon	COR						TER		
*Nephromatangeriense* (Maheu & A.Gillet) Zahlbr.	COR*	FLO				SJO		SMG	
*Ochrolechiaparella* (L.) A.Massal.	COR	FLO		PIC	GRA		TER		SMR
*Paralecanographagrumulosa* (Dufour) Ertz & Tehler	COR						TER	SMG	
*Parmotremacrinitum* (Ach.) M.Choisy 1952	COR		FAI	PIC	GRA	SJO	TER	SMG	
*Parmotremaperlatum* (Huds.) M.Choisy	COR	FLO	FAI	PIC	GRA	SJO	TER	SMG	SMR
*Parmotremareticulatum* (Taylor) M.Choisy	COR*	FLO	FAI	PIC	GRA	SJO	TER	SMG	SMR
*Peltigeracanina* (L.) Willd.	COR								
*Pertusariaficorum* Zahlbr.	COR								
*Physciaadscendens* H.Olivier	COR		FAI	PIC	GRA	SJO	TER	SMG	SMR
*Physciacaesia* (Hoffm.) Hampe ex Fürnr.	COR						TER	SMG	
*Porpidiaalbocaerulescens* (Wulfen) Hertel & Knoph.	COR							SMG	
*Porpidiacontraponenda* (Arnold) Knoph & Hertel 1984	COR			PIC	GRA		TER	SMG	
*Ramalinacanariensis* J.Steiner	COR		FAI				TER	SMG	SMR
*Ramalinacuspidata* (Ach.) Nyl.	COR*				GRA			SMG	
*Ramalinadecipiens* Mont. 1840	COR	FLO	FAI	PIC			TER	SMG	
*Ramalinarequienii* (De Not.) Jatta	COR		FAI	PIC	GRA	SJO	TER	SMG	SMR
*Ramalinasiliquosa* (Huds.) A.L.Sm.	COR	FLO	FAI	PIC		SJO		SMG	SMR
*Roccellaallorgei* Abbayes	COR*	FLO	FAI	PIC		SJO	TER	SMG	SMR
*Roccellafuciformis* (L.) DC.	COR		FAI	PIC	GRA	SJO	TER	SMG	SMR
*Roccellamaderensis* (J.Steiner) Follmann	COR*	FLO	FAI			SJO	TER	SMG	
*Roccellaphycopsis* Ach.	COR*	FLO	FAI	PIC	GRA	SJO	TER	SMG	SMR
*Roccellatinctoria* DC.	COR*	FLO	FAI	PIC	GRA	SJO	TER	SMG	SMR
*Roccellatuberculata* Vain.	COR*		FAI	PIC		SJO	TER	SMG	SMR
*Roccellographacircumscripta* (Leight.) Ertz & Tehler	COR*	FLO	FAI	PIC	GRA	SJO	TER	SMG	SMR
*Stereocaulonazoreum* (Schaer.) Nyl.	COR*	FLO	FAI	PIC		SJO	TER	SMG	
*Stereocaulonvesuvianum* Pers.	COR		FAI	PIC	GRA		TER	SMG	
*Teloschistesflavicans* (Sw.) Norman	COR	FLO	FAI	PIC	GRA	SJO	TER	SMG	SMR
*Trapeliainvoluta* (Taylor) Hertel	COR		FAI				TER		
*Trapeliaplacodioides* Coppins & P. James 1984	COR						TER		
***Usneacornuta*** Körb.	COR	FLO	FAI	PIC			TER	SMG	
*Usneasubflammea* P.Clerc	COR*		FAI	PIC			TER		
*Varicellarialactea* (L.) I.Schmitt & Lumbsch	COR				GRA		TER	SMG	
*Variosporaaurantia* (Pers.) Arup, Frödén & Søchting	COR						TER	SMG	
*Variosporaflavescens* (Huds.) Arup, Frödén & Søchting	COR		FAI		GRA		TER	SMG	
*Xanthoriaaureola* (Ach.) Erichsen	COR	FLO	FAI	PIC	GRA	SJO	TER	SMG	SMR
*Xanthoriaparietina* (L.) Th.Fr.	COR		FAI		GRA	SJO	TER	SMG	SMR
